# Crystal structure of bis­{*N*′-[(*E*)-4-hy­droxy­benzyl­idene]pyridine-4-carbohydrazide-κ*N* ^1^}di­iodidocadmium methanol disolvate

**DOI:** 10.1107/S2056989016019575

**Published:** 2017-01-01

**Authors:** Farhad Akbari Afkhami, Harald Krautscheid, Zeliha Atioğlu, Mehmet Akkurt

**Affiliations:** a Young Researchers and Elite Club, Tabriz Branch, Islamic Azad University, Tabriz, Iran; b Universität Leipzig, Fakultät für Chemie und Mineralogie, Johannisallee 29, D-04103 Leipzig, Germany; c İlke Education and Health Foundation, Cappadocia Vocational College, The Medical Imaging Techniques Program, 50420 Mustafapaşa, Ürgüp, Nevşehir, Turkey; d Department of Physics, Faculty of Sciences, Erciyes University, 38039 Kayseri, Turkey

**Keywords:** crystal structure, cadmium, hydrazone-based ligand, hydrogen bonding

## Abstract

In the title compound, [Cd(C_13_H_11_IN_3_O_2_)_2_]·2CH_3_OH, which crystallizes with *Z* = 4 in the space group *Pbcn*, the Cd^II^ atom is located on a twofold rotation axis and coordinated by two I^−^ anions and two N atoms from the pyridine rings of the two *N*′-[(*E*)-4-hy­droxy­benzyl­idene]pyridine-4-carbohydrazide ligands. The geometry around the Cd^II^ atom is distorted tetra­hedral, with bond angles in the range 94.92 (11)–124.29 (2)°. The iodide anions undergo inter­molecular hydrogen-bonding contacts with the C—H groups of the organic ligands of an adjacent complex mol­ecule, generating a chain structure along the *b* axis. Furthermore, an extensive series of O—H⋯O, N—H⋯O and C—H⋯O hydrogen-bonding inter­actions involving both the complex mol­ecules and the ethanol solvate mol­ecules generate a three-dimensional network.

## Abstract 2

In the title structure, the Cd^II^ atom is located on a twofold rotation axis and is coordinated by two I atoms and two N atoms of two carboxyl­ate groups of two planar *N*′-[(*E*)-4-hy­droxy­benzyl­idene]pyridine-4-carbohydrazide ligands. N—H⋯O, O—H⋯O, C—H⋯O and C—H⋯I hydrogen bonding assembles the mol­ecules into a three-dimensional network.

## Chemical context   

Hydrazones are organic compounds that incorporate –NH–N=CH– units in their mol­ecules. Hydrazone ligands based on pyridine are among the most important classes of flexible and versatile polydentate ligands and usually act as chelating ligands to metal cations (Afkhami *et al.*, 2016 ▸), but in some cases they behave as monodentate ligands through the pyridine group alone. The hydrazone-based ligand in the title compound was prepared according to a method reported in the literature (Deng *et al.*, 2005 ▸). The crystal structure of the ligand and three of its Zn^II^ metal complexes have been reported previously (Mahmoudi *et al.*, 2016 ▸). However, the title compound is the first reported crystal structure of a Cd^II^ complex of the ligand.
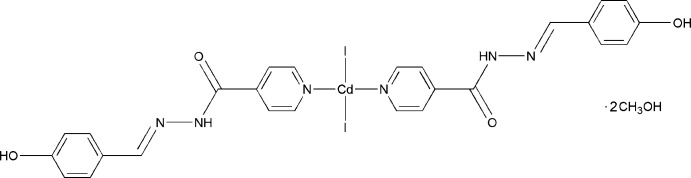


## Structural commentary   

**Figure 1:**
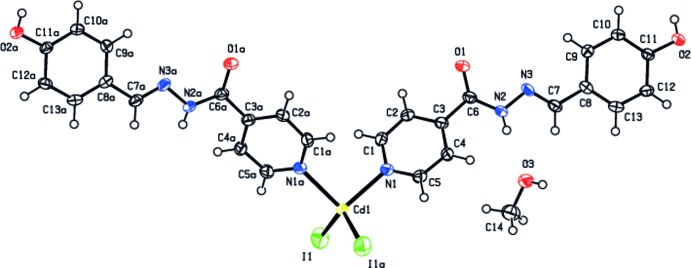
The molecular components of the title compound, showing the atom-numbering scheme. Displacement ellipsoids for non-H atoms are drawn at the 50% probability level [symmetry code: (*a*) −*x*, *y*, −*z* + 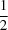].

The mol­ecular structure of the title compound is shown in Fig. 1 ▸. The Cd atom, located on a twofold rotation axis, is coordinated by two Schiff base ligands, acting as monodentate ligands, through the nitro­gen atoms of the pyridine rings. The angle between the benzene and pyridine rings is 35.42 (19)°. The Cd—I distance is 2.6909 (5) Å and the Cd—N distance is 2.297 (3) Å. All bonds and angles in the title compound fall within acceptable ranges and are comparable with those reported for related structures of bis­{2-[(2,4-di­methyl­phen­yl)imino­meth­yl]pyridine-κ^2^ *N*,*N*′}bis­(thio­cyanato-κ*N*)cadmium (Malekshahian *et al.*, 2012 ▸), di-μ-chlorido-bis­(chlorido­{*N*-[phen­yl(pyridin-2-yl-κ*N*)methyl­idene]pyridine-2-carbohydrazide-κ^2^ *N*,*O*}cadmium) (Akkurt *et al.*, 2014 ▸) and *cis*-di­aqua­bis[(*E*)-4-(2-hy­droxy­benzyl-idene­amino)­benzoato-κ^2^ *O*,*O*′]cadmium in which layers are built from strong O—H⋯O hydrogen bonding (Yao *et al.*, 2006 ▸).

## Supra­molecular features   

**Figure 2:**
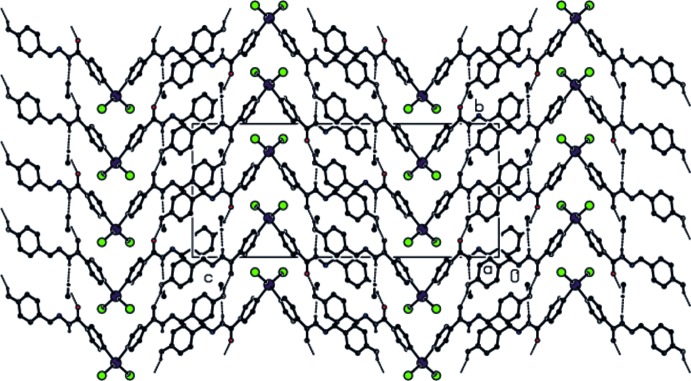
View of the hydrogen bonding and packing of the title compound along the *a* axis.

In the crystal, the iodide anions form inter­molecular C1—H1⋯I1 hydrogen-bonding contacts with the C—H groups of the pyridine rings of an adjacent complex mol­ecule. This generates a a chain structure along the *b* axis. In addition, an extensive series of O—H⋯O, N—H⋯O and C—H⋯O hydrogen-bonding inter­actions, Table 1 ▸, involving both the complex mol­ecules and the methanol solvate mol­ecules, generates a three-dimensional network (Figs. 2 ▸, 3 ▸ and 4 ▸).

## Synthesis and crystallization   

The title compound was synthesized by the reaction of a methanol solution of the ligand and Cd(NO_3_)_2_·4H_2_O in the presence of excess amount of NaI. The ligand (1 mmol, 0.240 g) and cadmium nitrate (1 mmol, 0.308 g) were placed in the main arm of a branched tube; sodium iodide (2 mmol, 0.300 g) was added to the mixture too. Methanol was carefully added to fill the arms. The tube was sealed and the ligand-containing arm was immersed in an oil bath at 333 K while the branched arm was kept at ambient temperature. After 24 h, suitable single crystals had deposited in the cooler arm which were isolated and air dried.

## Refinement   

Crystal data, data collection and structure refinement details are summarized in Table 2 ▸. All C-bound H atoms were ideal­ized (C—H = 0.98–0.99 Å) and refined using the riding-model approximation with *U*_iso_(H) = 1.2 or 1.5 *U*_eq_(C). The N—H and O—H hydrogen atoms were located from difference maps and refined with the restraints N2—H2*N* = 0.77 (5), O2—H2*O* = 0.81 (3), O3—H3*O* = 0.80 (3) Å and with *U*_iso_(H) = 1.2*U*_eq_(N) or *U*_iso_(H) = 1.5*U*_eq_(O).

## Supplementary Material

Crystal structure: contains datablock(s) global, I. DOI: 10.1107/S2056989016019575/sj5516sup1.cif

Structure factors: contains datablock(s) I. DOI: 10.1107/S2056989016019575/sj5516Isup2.hkl

CCDC reference: 1521096

Additional supporting information: crystallographic information; 3D view; checkCIF report

## Figures and Tables

**Figure 3:**
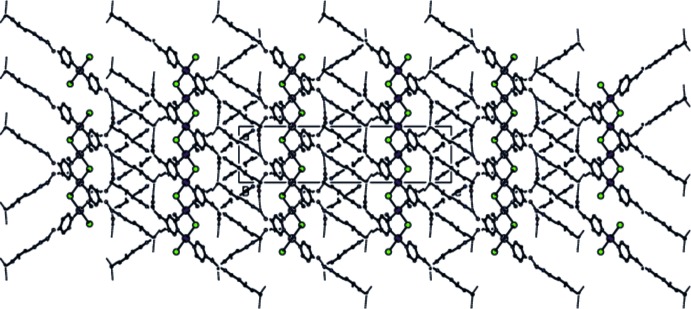
View of the hydrogen bonding and packing of the title compound along the *b* axis.

**Figure 4:**
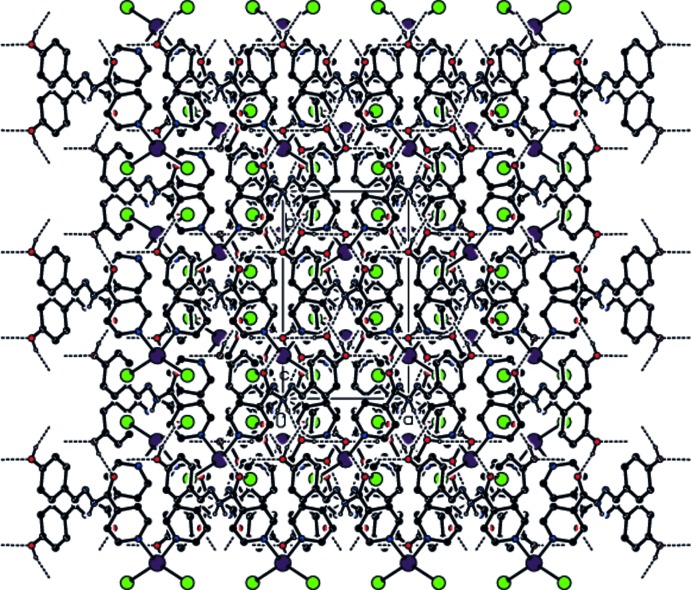
View of the hydrogen bonding and packing of the title compound along the *c* axis.

**Table 1:** Hydrogen-bond geometry (Å, °)

*D*—H⋯*A*	*D*—H	H⋯*A*	*D*⋯*A*	*D*—H⋯*A*
N2—H2*N*⋯O3	0.77 (5)	2.09 (5)	2.849 (4)	174 (4)
O2—H2*O*⋯O1^ii^	0.81 (3)	1.89 (4)	2.656 (4)	159 (5)
O3—H3*O*⋯O2^iii^	0.80 (3)	2.03 (2)	2.828 (5)	173 (4)
C4—H4⋯O3	0.94	2.58	3.291 (5)	133
C7—H7⋯O3	0.94	2.56	3.327 (5)	140
C1—H1⋯I1^iv^	0.94	3.11	3.811 (4)	133

Symmetry codes: (ii) 
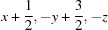
; (iii) 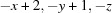; (iv) 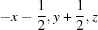.

**Table 2:** Experimental details

Crystal data	
Chemical formula	[Cd(C_13_H_11_IN_3_O_2_)_2_]·2CH_4_O
*M*_r_	912.79
Crystal system, space group	Orthorhombic, *P* *b* *c* *n*
Temperature (K)	213
*a*, *b*, *c* (Å)	8.0245 (4), 13.2482 (9), 30.4540 (19)
*V* (Å^3^)	3237.6 (3)
*Z*	4
Radiation type	Mo *K*α
μ (mm^−1^)	2.63
Crystal size (mm)	0.5 × 0.3 × 0.3

Data collection	
Diffractometer	Stoe IPDS1
Absorption correction	Numerical (*X-RED32*; Stoe & Cie, 2000 ▸)
*T*_min_, *T*_max_	0.337, 0.453
No. of measured, independent and observed [*I* > 2σ(*I*)] reflections	3511, 3511, 2542
(sin θ/λ)_max_ (Å^−1^)	0.651

Refinement	
*R*[*F* ^2^ > 2σ(*F* ^2^)], *wR*(*F* ^2^), *S*	0.040, 0.094, 0.88
No. of reflections	3511
No. of parameters	206
No. of restraints	2
H-atom treatment	H atoms treated by a mixture of independent and constrained refinement
Δρ_max_, Δρ_min_ (e Å^−3^)	1.68, −1.47

Computer programs: *EXPOSE*, *CELL*, *SELECT* and *INTEGRATE* (Stoe & Cie, 2000 ▸), *SHELXT2014* (Sheldrick, 2015*a* ▸), *SHELXL2014* (Sheldrick, 2015*b* ▸), *ORTEP-3 for Windows* and *WinGX* (Farrugia, 2012 ▸) and *PLATON* (Spek, 2009 ▸).

## supplementary crystallographic information

### Crystal data

**Table:**

[CdI_2_(C_13_H_11_N_3_O_2_)_2_]·2CH_4_O	*F*(000) = 1768
*M**_r_* = 912.79	*D*_x_ = 1.873 Mg m^−^^3^
Orthorhombic, *P**b**c**n*	Mo *K*α radiation, λ = 0.71073 Å
Hall symbol: -P 2n 2ab	Cell parameters from 428 reflections
*a* = 8.0245 (4) Å	θ = 2.9–27.6°
*b* = 13.2482 (9) Å	µ = 2.63 mm^−^^1^
*c* = 30.4540 (19) Å	*T* = 213 K
*V* = 3237.6 (3) Å^3^	Prism, colorless
*Z* = 4	0.5 × 0.3 × 0.3 mm

### Data collection

**Table:**

Stoe IPDS1 diffractometer	3511 independent reflections
Radiation source: fine-focus sealed tube	2542 reflections with *I* > 2σ(*I*)
Graphite monochromator	*R*_int_ = 0.000
phi scan	θ_max_ = 27.6°, θ_min_ = 2.9°
Absorption correction: numerical Stoe XRED32 (Stoe & Cie, 2000)	*h* = 0→9
*T*_min_ = 0.337, *T*_max_ = 0.453	*k* = 0→17
3511 measured reflections	*l* = 0→39

### Refinement

**Table:**

Refinement on *F*^2^	Hydrogen site location: mixed
Least-squares matrix: full	H atoms treated by a mixture of independent and constrained refinement
*R*[*F*^2^ > 2σ(*F*^2^)] = 0.040	*w* = 1/[σ^2^(*F*_o_^2^) + (0.0655*P*)^2^] where *P* = (*F*_o_^2^ + 2*F*_c_^2^)/3
*wR*(*F*^2^) = 0.094	(Δ/σ)_max_ < 0.001
*S* = 0.88	Δρ_max_ = 1.68 e Å^−^^3^
3511 reflections	Δρ_min_ = −1.47 e Å^−^^3^
206 parameters	Extinction correction: SHELXL-2014 (Sheldrick, 2015*b*), Fc^*^=kFc[1+0.001XFc^2^λ^3^/sin(2θ)]^-1/4^
2 restraints	Extinction coefficient: 0.0026 (2)

### Special details

**Table:**

Geometry. Bond distances, angles etc. have been calculated using the rounded fractional coordinates. All su's are estimated from the variances of the (full) variance-covariance matrix. The cell esds are taken into account in the estimation of distances, angles and torsion angles
Refinement. Refinement on F^2^ for ALL reflections except those flagged by the user for potential systematic errors. Weighted R-factors wR and all goodnesses of fit S are based on F^2^, conventional R-factors R are based on F, with F set to zero for negative F^2^. The observed criterion of F^2^ > 2sigma(F^2^) is used only for calculating -R-factor-obs etc. and is not relevant to the choice of reflections for refinement. R-factors based on F^2^ are statistically about twice as large as those based on F, and R-factors based on ALL data will be even larger.

### Fractional atomic coordinates and isotropic or equivalent isotropic displacement parameters (Å^2^)

**Table:**

	*x*	*y*	*z*	*U*_iso_*/*U*_eq_	
I1	−0.24029 (5)	0.11226 (3)	0.20424 (2)	0.0555 (1)	
Cd1	0.00000	0.20717 (2)	0.25000	0.0250 (1)	
O1	0.3473 (4)	0.62436 (18)	0.12617 (10)	0.0359 (9)	
O2	1.0000 (4)	0.70774 (18)	−0.10066 (9)	0.0310 (8)	
N1	0.1497 (5)	0.3244 (2)	0.21085 (10)	0.0284 (10)	
N2	0.5039 (5)	0.4973 (2)	0.09734 (10)	0.0263 (9)	
N3	0.5712 (5)	0.5598 (2)	0.06532 (10)	0.0269 (10)	
C1	0.0746 (6)	0.4087 (3)	0.19639 (12)	0.0295 (11)	
C2	0.1550 (5)	0.4791 (3)	0.17078 (12)	0.0280 (11)	
C3	0.3166 (5)	0.4612 (2)	0.15733 (12)	0.0247 (10)	
O3	0.6488 (4)	0.3011 (2)	0.09399 (12)	0.0431 (11)	
C4	0.3962 (6)	0.3747 (3)	0.17287 (13)	0.0302 (11)	
C5	0.3096 (6)	0.3093 (3)	0.19927 (13)	0.0319 (13)	
C6	0.3943 (5)	0.5354 (2)	0.12611 (12)	0.0262 (10)	
C7	0.6539 (5)	0.5138 (3)	0.03593 (12)	0.0265 (10)	
C8	0.7372 (5)	0.5675 (3)	−0.00022 (11)	0.0244 (10)	
C9	0.7230 (5)	0.6713 (3)	−0.00636 (13)	0.0290 (13)	
C10	0.8101 (6)	0.7194 (3)	−0.03945 (13)	0.0293 (13)	
C11	0.9119 (5)	0.6642 (2)	−0.06770 (11)	0.0230 (10)	
C12	0.9264 (6)	0.5601 (3)	−0.06211 (12)	0.0263 (10)	
C13	0.8388 (5)	0.5132 (3)	−0.02870 (12)	0.0268 (13)	
C14	0.5662 (7)	0.2065 (3)	0.09320 (17)	0.0450 (16)	
H1	−0.03730	0.42000	0.20410	0.0350*	
H2	0.10030	0.53890	0.16250	0.0340*	
H2N	0.541 (6)	0.444 (4)	0.0984 (14)	0.0320*	
H2O	0.967 (7)	0.765 (2)	−0.1033 (17)	0.0460*	
H4	0.50760	0.36150	0.16530	0.0360*	
H5	0.36440	0.25140	0.20980	0.0380*	
H7	0.66220	0.44310	0.03730	0.0320*	
H9	0.65340	0.70910	0.01220	0.0350*	
H10	0.80070	0.78960	−0.04290	0.0350*	
H12	0.99500	0.52210	−0.08090	0.0320*	
H13	0.84820	0.44290	−0.02520	0.0320*	
H3O	0.748 (3)	0.294 (5)	0.095 (2)	0.0650*	
H14A	0.62690	0.15860	0.11120	0.0670*	
H14B	0.56100	0.18190	0.06320	0.0670*	
H14C	0.45410	0.21430	0.10460	0.0670*	

### Atomic displacement parameters (Å^2^)

**Table:**

	*U*^11^	*U*^22^	*U*^33^	*U*^12^	*U*^13^	*U*^23^
I1	0.0416 (3)	0.0625 (2)	0.0624 (2)	−0.0191 (2)	0.0021 (2)	−0.0255 (2)
Cd1	0.0250 (2)	0.0179 (2)	0.0320 (2)	0.0000	0.0051 (2)	0.0000
O1	0.041 (2)	0.0198 (12)	0.0469 (16)	0.0042 (12)	0.0127 (14)	0.0091 (11)
O2	0.0326 (17)	0.0217 (11)	0.0386 (13)	0.0017 (13)	0.0098 (13)	0.0098 (10)
N1	0.035 (2)	0.0209 (13)	0.0292 (16)	0.0023 (13)	0.0071 (14)	0.0038 (12)
N2	0.031 (2)	0.0184 (12)	0.0294 (14)	0.0000 (14)	0.0067 (15)	0.0076 (11)
N3	0.027 (2)	0.0232 (13)	0.0306 (16)	−0.0034 (13)	0.0036 (14)	0.0093 (12)
C1	0.027 (2)	0.0294 (18)	0.0321 (19)	0.0061 (16)	0.0084 (16)	0.0039 (14)
C2	0.028 (2)	0.0216 (16)	0.0345 (19)	0.0068 (15)	0.0037 (16)	0.0054 (14)
C3	0.028 (2)	0.0198 (15)	0.0264 (17)	−0.0006 (14)	0.0006 (15)	0.0019 (12)
O3	0.031 (2)	0.0273 (14)	0.071 (2)	0.0040 (13)	0.0092 (16)	0.0070 (14)
C4	0.024 (2)	0.0267 (18)	0.040 (2)	0.0058 (15)	0.0078 (17)	0.0102 (15)
C5	0.034 (3)	0.0256 (18)	0.036 (2)	0.0079 (16)	0.0064 (17)	0.0066 (15)
C6	0.026 (2)	0.0202 (16)	0.0323 (18)	0.0002 (14)	−0.0008 (16)	0.0055 (13)
C7	0.028 (2)	0.0210 (16)	0.0304 (18)	−0.0032 (15)	0.0004 (16)	0.0062 (14)
C8	0.026 (2)	0.0224 (15)	0.0249 (16)	−0.0054 (15)	−0.0014 (15)	0.0041 (13)
C9	0.027 (3)	0.0241 (17)	0.0360 (19)	0.0039 (15)	0.0084 (16)	0.0031 (14)
C10	0.031 (3)	0.0180 (16)	0.039 (2)	0.0031 (15)	0.0057 (17)	0.0062 (14)
C11	0.022 (2)	0.0199 (15)	0.0271 (17)	−0.0009 (14)	−0.0007 (15)	0.0050 (13)
C12	0.032 (2)	0.0184 (15)	0.0285 (17)	0.0034 (15)	0.0018 (16)	0.0005 (13)
C13	0.033 (3)	0.0164 (15)	0.0310 (18)	−0.0025 (15)	−0.0038 (16)	0.0022 (13)
C14	0.044 (3)	0.036 (2)	0.055 (3)	−0.003 (2)	−0.003 (2)	0.003 (2)

### Geometric parameters (Å, º)

**Table:**

I1—Cd1	2.6909 (5)	C8—C13	1.391 (5)
Cd1—I1^i^	2.6909 (5)	C8—C9	1.393 (6)
Cd1—N1	2.297 (3)	C9—C10	1.382 (6)
Cd1—N1^i^	2.297 (3)	C10—C11	1.394 (5)
O1—C6	1.237 (4)	C11—C12	1.395 (5)
O2—C11	1.357 (4)	C12—C13	1.384 (6)
N1—C1	1.343 (5)	C1—H1	0.9400
N1—C5	1.346 (6)	C2—H2	0.9400
N2—N3	1.389 (4)	O3—H3O	0.80 (3)
N2—C6	1.340 (5)	C4—H4	0.9400
O2—H2O	0.81 (3)	C5—H5	0.9400
N3—C7	1.270 (5)	C7—H7	0.9400
C1—C2	1.376 (6)	C9—H9	0.9400
C2—C3	1.380 (6)	C10—H10	0.9400
N2—H2N	0.77 (5)	C12—H12	0.9400
C3—C4	1.395 (5)	C13—H13	0.9400
C3—C6	1.503 (5)	C14—H14A	0.9700
O3—C14	1.418 (5)	C14—H14B	0.9700
C4—C5	1.371 (6)	C14—H14C	0.9700
C7—C8	1.471 (5)		
			
I1—Cd1—N1	114.95 (8)	O2—C11—C12	117.9 (3)
I1—Cd1—I1^i^	124.29 (2)	O2—C11—C10	122.6 (3)
I1—Cd1—N1^i^	102.12 (9)	C10—C11—C12	119.5 (3)
I1^i^—Cd1—N1	102.12 (9)	C11—C12—C13	119.4 (4)
N1—Cd1—N1^i^	94.92 (11)	C8—C13—C12	121.6 (4)
I1^i^—Cd1—N1^i^	114.95 (8)	N1—C1—H1	119.00
Cd1—N1—C1	119.9 (3)	C2—C1—H1	119.00
Cd1—N1—C5	122.3 (2)	C1—C2—H2	120.00
C1—N1—C5	117.7 (3)	C3—C2—H2	120.00
N3—N2—C6	119.3 (3)	C14—O3—H3O	111 (5)
C11—O2—H2O	108 (4)	C5—C4—H4	121.00
N2—N3—C7	114.3 (3)	C3—C4—H4	120.00
N1—C1—C2	122.6 (4)	N1—C5—H5	119.00
C1—C2—C3	119.5 (4)	C4—C5—H5	119.00
C6—N2—H2N	125 (3)	C8—C7—H7	119.00
N3—N2—H2N	115 (3)	N3—C7—H7	119.00
C2—C3—C4	118.1 (3)	C8—C9—H9	120.00
C2—C3—C6	117.7 (3)	C10—C9—H9	120.00
C4—C3—C6	124.2 (4)	C11—C10—H10	120.00
C3—C4—C5	119.1 (4)	C9—C10—H10	120.00
N1—C5—C4	122.9 (4)	C11—C12—H12	120.00
N2—C6—C3	116.1 (3)	C13—C12—H12	120.00
O1—C6—C3	119.7 (3)	C8—C13—H13	119.00
O1—C6—N2	124.0 (3)	C12—C13—H13	119.00
N3—C7—C8	122.2 (4)	O3—C14—H14A	110.00
C7—C8—C9	122.7 (3)	O3—C14—H14B	109.00
C7—C8—C13	118.9 (4)	O3—C14—H14C	109.00
C9—C8—C13	118.4 (3)	H14A—C14—H14B	110.00
C8—C9—C10	120.8 (4)	H14A—C14—H14C	109.00
C9—C10—C11	120.3 (4)	H14B—C14—H14C	109.00
			
I1—Cd1—N1—C1	66.6 (3)	C2—C3—C6—O1	27.7 (5)
I1^i^—Cd1—N1—C1	−156.1 (3)	C2—C3—C4—C5	2.5 (6)
N1^i^—Cd1—N1—C1	−39.2 (3)	C6—C3—C4—C5	−176.2 (4)
I1—Cd1—N1—C5	−109.3 (3)	C2—C3—C6—N2	−147.5 (4)
I1^i^—Cd1—N1—C5	28.1 (3)	C4—C3—C6—O1	−153.5 (4)
N1^i^—Cd1—N1—C5	144.9 (3)	C3—C4—C5—N1	0.4 (6)
C1—N1—C5—C4	−1.5 (6)	N3—C7—C8—C9	−4.1 (6)
Cd1—N1—C1—C2	−176.4 (3)	N3—C7—C8—C13	173.8 (4)
C5—N1—C1—C2	−0.4 (6)	C7—C8—C9—C10	176.7 (4)
Cd1—N1—C5—C4	174.5 (3)	C13—C8—C9—C10	−1.2 (6)
C6—N2—N3—C7	−170.1 (4)	C7—C8—C13—C12	−177.1 (4)
N3—N2—C6—O1	0.2 (6)	C9—C8—C13—C12	0.9 (6)
N3—N2—C6—C3	175.1 (3)	C8—C9—C10—C11	1.0 (6)
N2—N3—C7—C8	−178.8 (4)	C9—C10—C11—O2	−180.0 (4)
N1—C1—C2—C3	3.3 (6)	C9—C10—C11—C12	−0.5 (6)
C1—C2—C3—C6	174.6 (3)	O2—C11—C12—C13	179.8 (4)
C1—C2—C3—C4	−4.3 (5)	C10—C11—C12—C13	0.3 (6)
C4—C3—C6—N2	31.2 (5)	C11—C12—C13—C8	−0.5 (6)

Symmetry code: (i) −*x*, *y*, −*z*+1/2.

### Hydrogen-bond geometry (Å, º)

**Table:**

*D*—H···*A*	*D*—H	H···*A*	*D*···*A*	*D*—H···*A*
N2—H2*N*···O3	0.77 (5)	2.09 (5)	2.849 (4)	174 (4)
O2—H2*O*···O1^ii^	0.81 (3)	1.89 (4)	2.656 (4)	159 (5)
O3—H3*O*···O2^iii^	0.80 (3)	2.03 (2)	2.828 (5)	173 (4)
C4—H4···O3	0.94	2.58	3.291 (5)	133
C7—H7···O3	0.94	2.56	3.327 (5)	140
C1—H1···I1^iv^	0.94	3.11	3.811 (4)	133

Symmetry codes: (ii) *x*+1/2, −*y*+3/2, −*z*; (iii) −*x*+2, −*y*+1, −*z*; (iv) −*x*−1/2, *y*+1/2, *z*.
